# Exposure to silver and titanium dioxide nanoparticles at supra-environmental concentrations decreased sperm motility and affected spermatozoa subpopulations in gilthead seabream, *Sparus aurata*

**DOI:** 10.1007/s10695-023-01218-0

**Published:** 2023-07-12

**Authors:** Catarina C.V. Oliveira, Leonor Ferrão, Victor Gallego, Cláudia Mieiro, Isabel B. Oliveira, Ana Carvalhais, Mário Pachedo, Elsa Cabrita

**Affiliations:** 1 Centre of Marine Sciences (CCMAR), University of Algarve, 8005-139 Faro, Portugal; 2 Aquaculture and Biodiversity Group, Universitat Politècnica de València, 46022 València, Spain; 3 Centre for Environmental and Marine Studies (CESAM) and Department of Biology, University of Aveiro, 3810-193 Aveiro, Portugal

**Keywords:** Nanoparticles, Reprotoxicity, Ex vivo exposure, Sperm subpopulations

## Abstract

Marine pollution by nanoparticles (NPs) can be reprotoxic for fish and disturb successful reproduction of wild populations. In gilthead seabream (*Sparus aurata*), a mild effect on sperm motility was observed after exposure to high concentrations of silver NPs. Considering the great heterogeneity traits within a sperm sample, it is possible that NPs affect spermatozoa accordingly, modulating subpopulation profile. Thus, this work aimed to analyse NP effects in sperm motility in general and considering spermatozoa population structure, using a subpopulation approach. Seabream sperm samples from mature males were exposed for 1 h to increasing concentrations of titanium dioxide (1, 10, 100, 1000 and 10,000 μg L^−1^) and silver (0.25, 25 and 250 μg L^−1^) NPs, including Ag NP and Ag^+^, dissolved in a non-activating medium (0.9 % NaCl). Concentrations chosen include realistic (10–100 and 0.25 μg L^−1^, respectively, for TiO_2_ and Ag) and supra-environmental values. The mean particle diameter was determined as 19.34 ± 6.72 and 21.50 ± 8.27 nm in the stock suspension, respectively, for titanium dioxide and silver. After the ex vivo exposure, sperm motility parameters were determined using computer-assisted sperm analysis, and sperm subpopulations were later identified using a two-step cluster analysis. Results revealed a significant reduction in total motility after exposure to the 2 highest concentrations of titanium dioxide NPs, while curvilinear and straight-line velocities were not altered. Exposure to silver NPs (Ag NP and Ag^+^) lowered significantly total and progressive motilities at all concentrations, while curvilinear and straight-line velocities were significantly lower only at the highest concentration. Sperm subpopulations were also affected by the exposure to both titanium dioxide and silver NPs. In both cases, the highest levels of NPs triggered a decrease in the percentage of fast sperm subpopulations (38.2% in TiO_2_ 1000 μg L^−1^, 34.8.% in Ag NP 250 μg L^−1^, and 45.0% in Ag^+^ 250 μg L^−1^ vs 53.4% in the control), while an increase on slow sperm subpopulations. A reprotoxic effect was proven for both NPs, but only at supra-environmental concentrations.

## Introduction

Nanotechnology is an area with exceptional potential, thus being a developing field of scientific and industrial interest worldwide. In consequence, the commercialization of nanoparticles (NPs) has also been rapidly expanding, with applications for industry, medicine and many types of consumer products. Incorporation of these nanomaterials into a range of manufactured goods, raises an increasing concern about the subsequent release of NPs into the environment, namely aquatic systems (Asztemborska et al. 2018). Among the most used NPs, titanium dioxide NP (TiO_2_ NP) and silver NP (Ag NP) are present for example in sunscreens, cosmetics, dyes, antimicrobial products, whose production corresponds to 89–97% of the total NP emissions into the environment. These NPs are considered the most relevant concerning human and environmental hazards (Keller et al. 2013). In the aquaculture field, nanotechnology has also found several direct (e.g. feeding industry and fish disease control) and indirect (e.g. wastewater treatment, fishpond sterilization and packaging) applications, but their implications are still unknown. Khosravi-Katuli et al. (2017) reviewed the applications of NPs in aquaculture, including TiO_2_ and Ag NPs, and pointed the main negative effects observed in commercial species, including fish, bivalves and crustaceans. Effects range from increases in ROS production, lipid peroxidation, apoptosis and DNA damage to alteration of the antioxidant enzymes, increased malformation of larvae and even mortality (Khosravi-Katuli et al. 2017). However, these effects are not restricted to aquaculture fish. When nanoparticles from consumer products, industry or aquaculture usages leach into the ocean, they can induce a range of similar toxic effects in marine organisms, which can include adverse non-hereditary effects in reproduction, or reprotoxic, and thus disturb the successful reproduction of wild populations (Kurwadkar et al. 2015).

Recent investigation performed on an aquaculture commercial species, gilthead seabream (*Sparus aurata*), has proven a mild effect in sperm motility, after short-term exposure to high concentrations of Ag NPs, while TiO_2_ did not produce any effect, considering sperm as a homogenous population (Carvalhais et al. 2022). However, several studies indicated that there is a great diversity in spermatozoa within a same sperm sample, which could mask the NP effect. This variability has been explained by the presence of different sperm subpopulations within a sperm sample, which differs mainly in motility and morphology, but may result from differences in physiological and molecular mechanisms (Martínez-Pastor 2022). The most common method used to measure sperm motility is the CASA system that can give information on the mean motility parameters of an examined field or can give information on the kinematic parameters of each spermatozoon. Usually, researchers mostly rely on the mean values, considering the whole semen sample as homogeneous and not taking advantage of the amount of data available. When analysing the whole data, it is possible to obtain much more information about the heterogeneity of a sperm sample. Applying subpopulation analysis to such data allows for the analysis of groups of spermatozoa with similar motility features and to estimate sperm quality for each male (Martínez-Pastor et al. 2011; Gil Anaya et al. 2015).

The presence of sperm subpopulations has already been identified in a variety of mammal species (Caldeira et al. 2018), including human (Santolaria et al. 2016), in reptiles (American crocodile, *Crocodylus acutus*, Valverde et al. (2021)) and birds (rooster, *Gallus domesticus* and Guinea fowl, *Numida meleagris*, García-Herreros (2016)). In fish, also a few papers described different populations in sperm samples, in species such as the *Senegalese sole*, *Solea senegalensis* (Martínez-Pastor et al. 2008), steelhead, *Oncorhynchus mykiss* (Kanuga et al. 2012), the Atlantic salmon, *Salmo salar* (Kanuga et al. 2012), the European eel, *Anguilla anguilla* (Gallego et al. 2015), the tambaqui, *Colossoma macropomum* (Gallego et al. 2017), and in our model species, the gilthead seabream (Beirão et al. 2011). Beirão et al. (2011) identified three different sperm subpopulations that were correlated differently with embryo hatching rates. These authors also proved that sperm composition in terms of subpopulations was differentially affected by cryopreservation procedures, with the “fast linear spermatozoa” being the best-represented subpopulation at the beginning of motility activation and the ones showing a greater decrease in time post-activation. In this sense, it is possible that NP toxicity could also affect the sperm population according to their heterogeneity traits, modulating subpopulation profile. Thus, in the current work, we aimed to analyse the effect of TiO_2_ and Ag NPs in all spermatozoa population structures and using a subpopulation approach. With this purpose, environmentally relevant and supra-environmental concentrations of TiO_2_ and Ag NPs (including Ag^+^) were used on the sperm of gilthead seabream (*Sparus aurata*), upon a short-term incubation with NP (ex vivo).

## Materials and methods

### Sperm sample collection

Sperm samples used in the ex vivo trials were obtained from sexually mature gilthead seabream (*Sparus aurata*) males (2+ years old, approximately 0.5 kg), provided by Aqualvor Lda fish farm (Lagos, Portugal), during their reproductive season (December–February in the South of Portugal). Before sperm collection, gentle abdominal pressure was applied to eliminate urine in the ducts, and the urogenital pore was cleaned to eliminate seawater, mucus or faeces residues. Sperm was then collected by aspirating with a 1-ml syringe without a needle, while applying a gentle abdominal massage to release it. Samples were kept on ice in Eppendorf tubes, in a polystyrene support to avoid direct contact with ice (approximately at 10 °C), until analysis. All samples showing urine contamination were rejected. A total of 30 sperm samples (1 ml volume, mean total motility 85.57 ± 4.86 %), corresponding to different individual males, were collected for each trial.

### Experimental design

To evaluate the reprotoxic effect of TiO_2_ and Ag NP (including Ag^+^) on sperm motility, sperm exposure to these compounds was performed ex vivo. Several concentrations were tested in two independent trials: TiO_2_ NP tested concentrations reflected the levels found in Mediterranean marine waters (water column and top surface layer: 10 to 100 μg L^−1^) (Labille et al. 2020), as well as supra-environmental concentrations. Ag NP levels replicated the levels found in French wastewater (influent and effluent: 0.25 μg L^−1^) (Deycard et al. 2017) and supra-environmental concentrations. Five concentrations of TiO_2_ NP (10, 100, 1000, 10,000 and 100,000 μg L^−1^) and three concentrations of Ag NP (0.25, 25 and 250 μg L^−1^) were tested. To evaluate the effects of Ag NP oxidative dissolution in water (Zhang et al. 2016), the same concentrations of Ag+ (0.25, 25 and 250 μg L^−1^) were also assessed estimating the contribution of the dissolved Ag form. The same sperm samples were exposed to 0.9% NaCl, to be used as a control, in all trials. In the ex vivo exposures, sperm was diluted in a proportion of 1:10 (v/v) in the exposure medium (0.9% NaCl with NP), respectively, to achieve a final sperm concentration of ~ 2×10^7^ cells ml^−1^. Exposure lasted for 1 h, with gentle agitation performed every 15 min, and was performed at 10 °C to avoid sperm degradation (Gallego and Asturiano 2019). After exposure, sperm was used for the evaluation of sperm motility.

### Chemicals utilized and preparation of solutions

The preparation of the NP suspensions for the sperm exposure assays was performed as previously described by (Carvalhais et al. 2022). The chemicals used in the preparation of the NP suspensions were all provided by Sigma-Aldrich/Merck. For TiO_2_ NP (Aeroxide®P25, purity ≥99.5% CAS# 13463–67-7), firstly a stock solution (1×10^6^ μg L^−1^) was prepared in ultra-pure water, by sonication with an ultrasonic processor (Q125 Sonicator®, QSonica), for 15 min at 125 W, with 5:1 pulses on/off, and in an ice bath to avoid overheating. Afterward, TiO_2_ NP working solutions (10, 100, 1000, 10,000 and 100,000 μg L^−1^) were prepared by successive dilutions of the stock, in a sterile non-activated medium, namely saline solution (0.9 % NaCl).

To test the effect of Ag NPs, silver dispersion in an aqueous buffer (2×10^4^ μg L^−1^, ref. 730785) was used. A 2500 μg L^−1^ stock solution was prepared in ultra-pure water and the working suspensions (0.25, 25 and 250 μg L^−1^) in 0.9 % NaCl by successive dilutions of the stock. To evaluate the potential effect of Ag ion (Ag^+^) on the measured endpoints, Ag^+^ solutions were prepared using AgNO_3_ (purity ≥99%, ACS reagent, CAS# 7761-88-8). AgNO_3_ stock solutions of 4000 μg L^−1^ ([Ag+] = 2500 μg L^−1^) were prepared in ultra-pure water, and the working solutions (0.40, 40, 400 μg L^−1^, [Ag+] = 0.25, 25 and 250 μg L^−1^) were prepared in 0.9 % NaCl, by successive dilutions of the stock. Stock and working suspensions were always vortexed before the next dilution. We will refer to the use of Ag+ present in AgNO_3_ as Ag+, in contrast to Ag NP.

Particle size and dispersion from NP suspensions were confirmed by scanning transmission electron microscopy (STEM), and size distribution was assessed by dynamic light scattering (DLS) analysis, as reported by Carvalhais et al. (2022).

### Sperm motility analysis

Spermatozoa motility was assessed using the CASA system (ISAS - Integrated System for Sperm Analysis; Proiser, Valencia, Spain) coupled to a phase-contrast microscope (Nikon E-200; Nikon, Tokyo, Japan) with an ISAS camera (Gallego and Asturiano 2018; Gallego and Asturiano 2019). For sperm activation, 10 μL of artificial seawater (NaCl, Sigma S9888,1100 mOsm kg^−1^) was added to 1 μL of the cell suspension in a Makler chamber, and motility was recorded at 15, 30, 45 and 60 s post-activation, always in the same field. Image sequences were captured with a 10× negative phase contrast objective, saved and analysed afterwards using the ISAS software. The software settings were adjusted to gilthead seabream sperm, using 25 frames per second and considering the head area of 1 to 90 μm^2^, as previously adapted for this species (Cabrita et al. 2011). The CASA software parameters registered for the evaluation of spermatozoa motility were percentage of motile cells (total motility, TM; %), percentage of progressive cells (progressive motility, PM; %), velocity according to the actual path (Curvilinear velocity, VCL; μm s^−1^), velocity according to the straight path (straight-line velocity, VSL; μm s^−1^) and linearity (LIN; %) (Gallego et al. 2014). Besides these parameters, average path velocity (VAP; μm s^−1^), straightness (STR; %), wobble coefficient (WOB; %), mean amplitude of lateral head displacement (ALH; μm) and frequency of head displacement (BCF; Hz) were also considered for sperm subpopulation characterization. Spermatozoon was considered motile when VCL was higher than 10 μm s^−1^. Samples with a TM < 45% in the control were discarded, resulting in a final number of 21 samples exposed to each treatment.

### Statistical and sperm subpopulation analysis

Statistical analyses and data plotting were performed using Microsoft Excel®, and IBM® SPSS Statistics 29. Motility parameters are represented as the mean ± standard error of the means (SEM), at each post-activation time. A general linear model with the Bonferroni correction was used (*p* < 0.05) to analyse all sperm motility parameters. Motility data sets were tested for equality of variances using Levene’s test (Zar 1999), and transformed data was used, whenever this assumption was not confirmed. Statistical comparisons were then performed using the whole curve of motility recorded time (until 60 s), comparing the tendency of the curves for each NP concentration (Martínez-Páramo et al. 2013).

For sperm subpopulation analysis, eight kinetic parameters obtained with the CASA system were used, considering each spermatozoon: VCL, VSL, VAP, STR, LIN, WOB, ALH and BCF. Data from all motile spermatozoa (obtained both from control and exposed NPs samples) were imported into a single dataset, which represented approximately 51.150 spermatozoa (removing firstly the immotile spermatozoa (VCL < 10 μm s^−1^) and any obvious outliers). Control samples from all trials were pooled together in a unique control. The two-step cluster analysis procedure is an exploratory tool designed to reveal natural groupings (or clusters) within a data set that would otherwise not be apparent. Clustering was carried out using the log-likelihood distances and Schwarz’s Bayesian criterion (BIC).

## Results

### Characterization of the nanoparticle’s suspensions

STEM confirmed the standard spheroid irregular shape of TiO_2_ NP and a primary size of a mean particle diameter of 19.34 ± 6.72 nm in the stock suspension. DLS analysis of both stock and working suspensions of TiO_2_ NP displayed the presence of agglomerates. For Ag NP, STEM revealed a spherical nature with a mean primary size particle diameter of 21.50 ± 8.27 nm. DLS analysis of Ag NP (stock and working suspensions) showed the presence of agglomerates.

### Sperm motility analysis

**Fig. 1:**
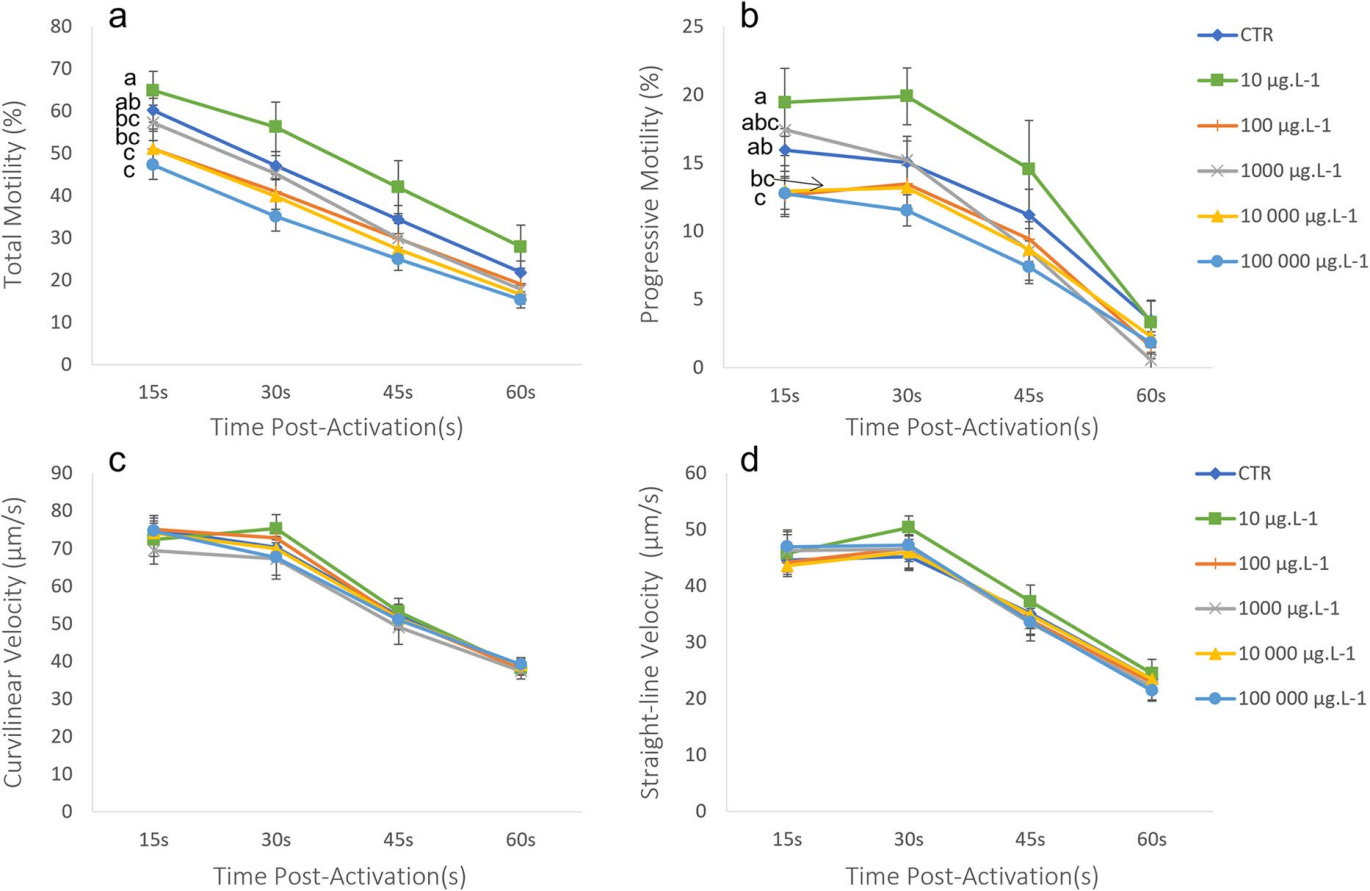
Gilthead seabream sperm motility (*n*=21) registered after 1 h of exposure to titanium dioxide nanoparticles (TiO_2_ NP: 10, 100, 1000, 10,000 and 100,000 μg L^−1^). A control exposure to the same solution without TiO_2_ was also performed. Values are represented by mean ± SEM, for each post-activation time (15, 30, 45 and 60 s). Parameters measured include **A** total motility, **B** progressive motility, **C** curvilinear velocity, and **D** straight-line velocity. Different letters indicate significant differences between TiO_2_ NP concentrations. Statistical analysis was performed with the whole curve during the whole motility recorded time (general linear model, Bonferroni, *p* < 0.05)

**Fig. 2:**
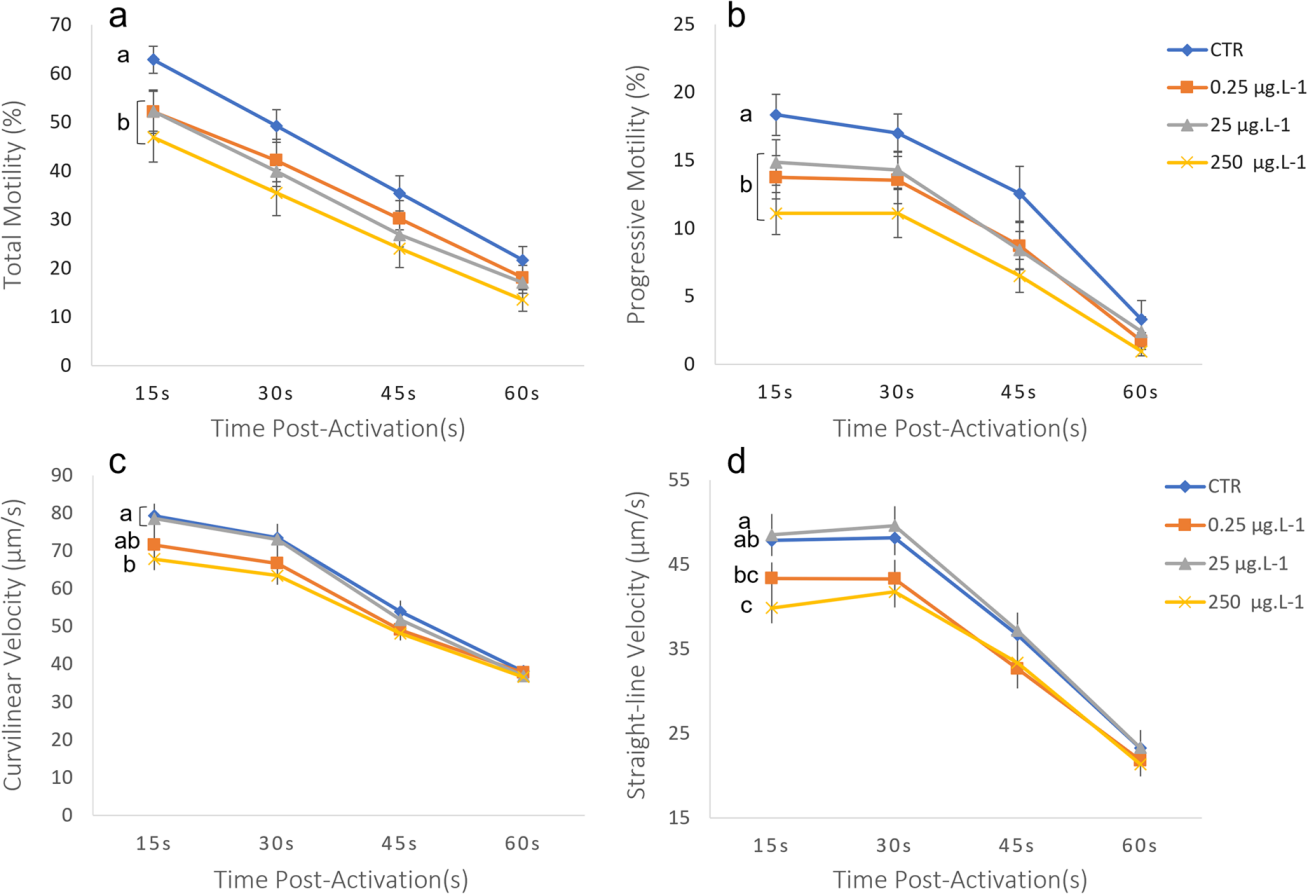
Gilthead seabream sperm motility (*n*=21) registered after 1 h of exposure to silver nanoparticles (Ag NP: 0.25, 25 and 250 μg L^−1^). A control exposure to the same solution without Ag NP was also performed. Values are represented by mean ± SEM, for each post-activation time (15, 30, 45 and 60 s). Parameters measured include **A** total motility, **B** progressive motility, **C** curvilinear velocity and **D** straight-line velocity. Different letters indicate significant differences between Ag NP concentrations. Statistical analysis was performed with the whole curve during the whole motility recorded time (general linear model, Bonferroni, *p* < 0.05)

**Fig. 3:**
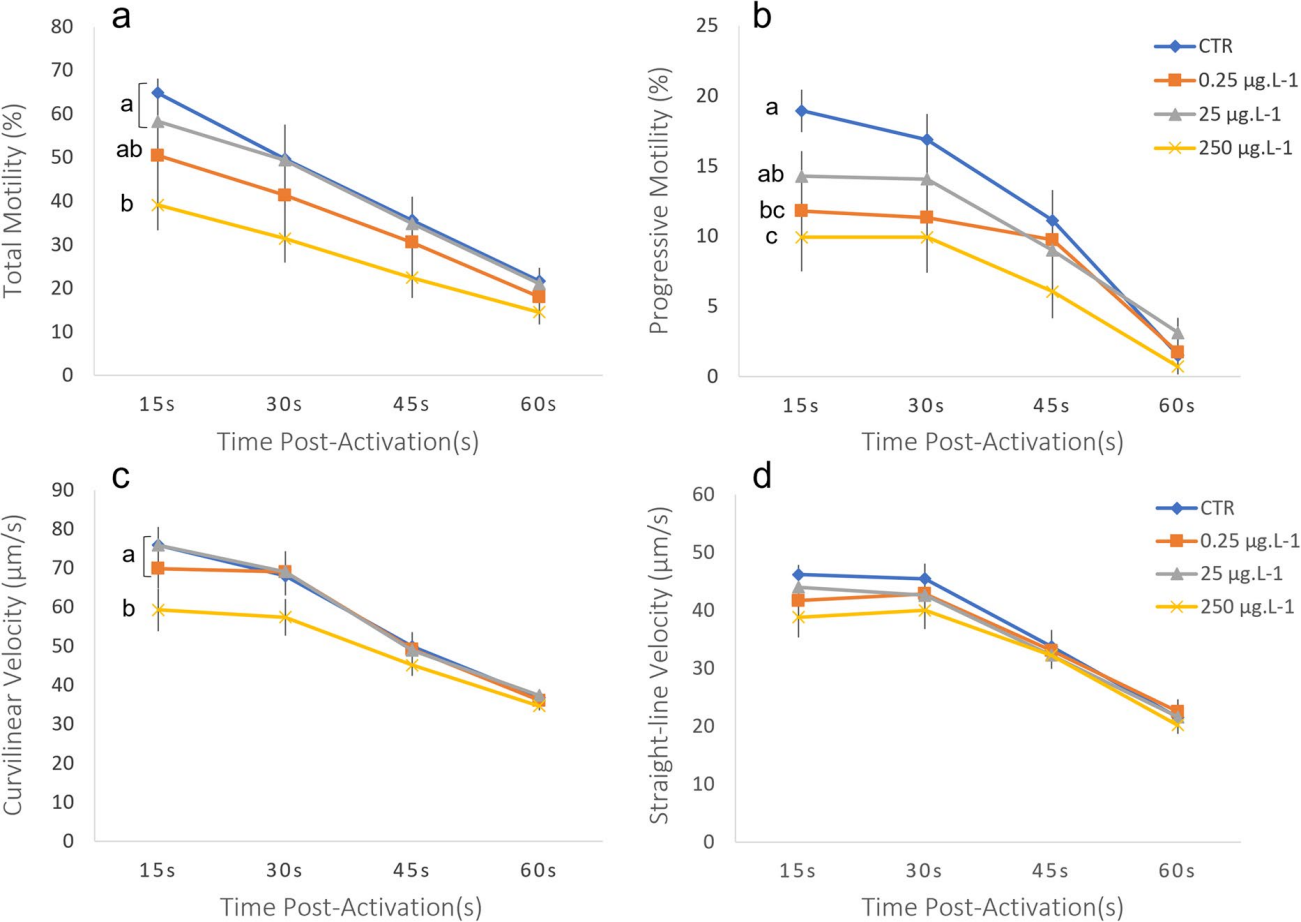
Gilthead seabream sperm motility (*n*=21) registered after 1 h of exposure to silver ion (Ag^+^: 0.25, 25 and 250 μg L^−1^). A control exposure to the same solution without Ag^+^ was also performed. Values are represented by mean ± SEM, for each post-activation time (15, 30, 45 and 60 s). Parameters measured include **A** total motility, **B** progressive motility, **C** curvilinear velocity and **D** straight-line velocity. Different letters indicate significant differences between AgNo_3_ concentrations. Statistical analysis was performed with the whole curve during the whole motility recorded time (general linear model, Bonferroni, *p* < 0.05)

As seen in Figs. 1, 2 and 3, both TiO_2_ and Ag (including Ag^+^) NPs affected sperm motility negatively, but only at the highest concentrations. In the case of TiO_2_ (Fig. 1), TM was significantly lowered in comparison to the control, after the exposure to concentrations of 10,000 and 100,000 μg L^−1^. The lowest concentration, 10 μg L^−1^, had a similar TM in comparison with the control, and significantly higher values when compared to all other concentrations (100, 1000, 10,000 and 100,000 μg L^−1^). In terms of PM, the results were similar. PM values were significantly lower in relation to the control after the exposure to the highest concentration, 100,000 μg L^−1^, while concentrations of 100 and 10,000 μg L^−1^ resulted in lower PM values, but in relation to the lowest TiO_2_ NP concentration, 10 μg L^−1^. This concentration presented statistically similar PM values to the control, as observed for the TM. The spermatozoa velocities, VCL and VSL, were not affected by any of the concentrations of TiO_2_ NP.

For Ag, an effect in sperm motility was seen both for Ag NP and Ag+ ion. The exposure to Ag NP provoked a significant decrease in the percentage of both TM and PM, for all three concentrations tested (Fig. 2). In terms of velocities, only the highest concentration (250 μg L^−1^) reduced significantly the VCL and VSL in comparison with the control. When sperm was exposed for 1 h to increasing concentrations of Ag+ (Fig. 3), TM was only significantly lower at 250 μg L^−1^ and PM decreased under concentrations of 0.25 and 250 μg L^−1^. VCL was significantly lower than the control at the highest concentration, and VSL was not affected.

Linearity was the only motility parameter unaffected by any of the NP concentrations tested.

### Spermatozoa subpopulations

The application of a two-step cluster analysis to the values obtained by ISAS software rendered from the control and treated samples, yielded four sperm subpopulations (SP1, SP2, SP3 and SP4), which were characterized by the mean values of kinetic parameters (Table 1).

**Table 1:** Mean values ± SD obtained from the clustering analysis for the sperm subpopulations (SP1, SP2, SP3 and SP4) of control and exposed samples, based on the kinetic parameters given by CASA system (*n*=21 males; *n*=51.150 spermatozoa). CASA system kinetic parameters include the following: *VCL*, curvilinear velocity; *VSL*, straight-line velocity; *VAP*, average path velocity; *LIN*, linearity; *STR*, straightness; *WOB*, wobble; *ALH*, amplitude of lateral head displacement; *BCF*, beat cross frequency. Also, total percentage encountered is given for each SP

Kinetic parameters	SP1	SP2	SP3	SP4
VCL (μm s^−1^)	103.1 ± 23.1	102.1 ± 21.3	35.4 ± 16.3	34.3 ± 14.8
VSL (μm s^−1^)	78.4 ± 21.1	32.8 ± 15.1	24.0 ± 12.4	9.2 ± 5.1
VAP (μm s^−1^)	95.1 ± 22.2	81.4 ± 20.6	30.4 ± 14.6	23.2 ± 11.8
LIN (%)	76.8 ± 14.2	32.4 ± 13.8	67.3 ± 14.1	28.0 ± 11.4
STR (%)	82.9 ± 12.6	41.2 ± 18.2	78.5 ± 12.4	43.5 ± 19.2
WOB (%)	92.3 ± 6.2	79.6 ± 9.7	85.6 ± 9.6	66.7 ± 13.4
ALH (μm)	2.0 ± 0.6	3.0 ± 0.8	1.4 ± 0.4	1.7 ± 0.5
BCF (Hz)	6.3 ± 2.4	4.9 ± 2.3	4.4 ± 2.4	3.9 ± 2.1
Total %	23.1	22.6	33.4	20.9

The first subpopulation (SP1) included spermatozoa with high values of VCL, LIN and STR; hence, they were labelled fast and linear spermatozoa. The SP1 represented 23.1% of the total analysed spermatozoa. The second subpopulation (SP2) included spermatozoa with high values of VCL and low values of LIN and STR, so they were labelled as fast nonlinear spermatozoa. This subpopulation (SP2) represented the 22.6% of the total analysed spermatozoa. The third subpopulation (SP3) included spermatozoa with low values of VCL and high values of LIN and STR, then they were labelled as slow linear spermatozoa. That subpopulation (SP3) was the most abundant and represented the 33.4% of the total analysed spermatozoa. Finally, the fourth subpopulation (SP4) included spermatozoa with low values of VCL, LIN and STR, and they were labelled as slow nonlinear spermatozoa. That subpopulation (SP4) was the less abundant, representing the 20.9% of the total analysed spermatozoa.

**Fig. 4:**
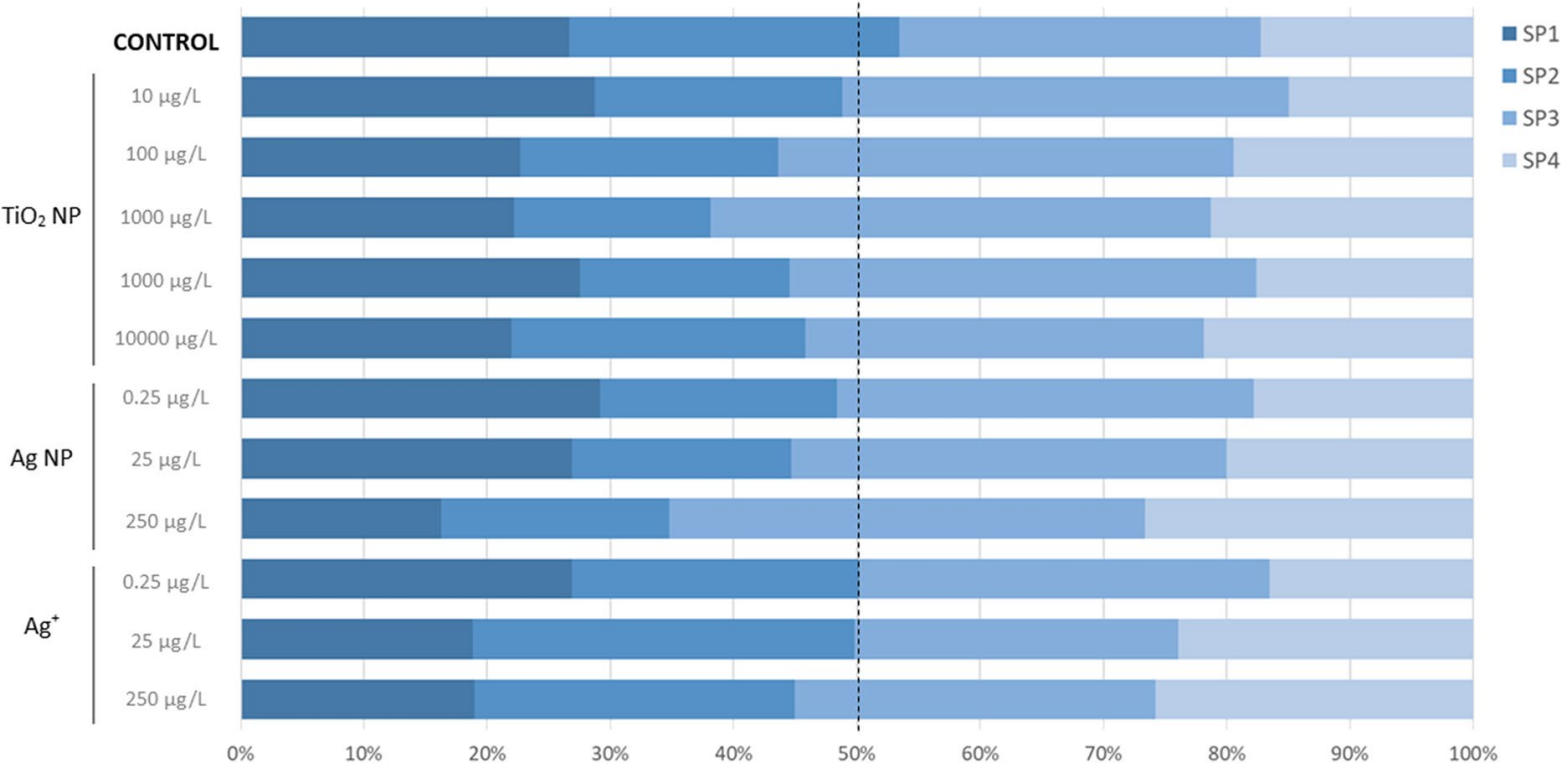
Percentage of spermatozoa in each sperm subpopulations (SP1, SP2, SP3 and SP4) of gilthead seabream samples (*n*=21) exposed for 1 h to titanium dioxide nanoparticles (TiO_2_ NP: 10, 100, 1000, 10,000 and 100,000 μg L^−1^), to silver nanoparticles (Ag NP: 0.25, 25 and 250 μg L^−1^), silver ion (Ag^+^: 0.25, 25 and 250 μg L^−1^) and control

Regarding the study of sperm subpopulations according to the exposure to the different NPs, the results are shown in Fig 4. In that sense, and taking as reference the fast sperm subpopulations detected in this study (SP1 and SP2), data showed that the percentage of SP1 and SP2 on the control group was 53.4%.

However, as the sperm was exposed to different nanoparticles at increasing concentrations, the percentage of the fast sperm subpopulations (SP1 and SP2) was decreasing until reaching minimum values of 38.2% in TiO_2_ NP (1000 μg L^−1^), 34.8.% in Ag NP (250 μg L^−1^), and 45.0% in Ag^+^ (250 μg L^−1^)

## Discussion

In the present work, a TiO_2_ and Ag NPs reprotoxic effect was demonstrated for gilthead seabream sperm after ex vivo exposure to the highest concentrations tested. This effect was observed both considering sperm as a homogenous sample or having in mind the different sperm subpopulations present in an ejaculate. The reprotoxic effect of these marine contaminants is poorly studied in fish sperm and has never been tested on sperm subpopulations. The results herein obtained showed the detrimental effect supra-environmental concentrations of TiO_2_ and realistic concentrations of Ag NP can have on sperm motility in gilthead seabream, thus suggesting the importance of monitoring their levels in the sea.

When sperm was exposed for 1 h to increasing concentrations of TiO_2_ NP, total and progressive motilities (TM and PM) were negatively affected by concentrations of 10,000 and 100,000 μg L^−1^ and 100,000μg L^−1^, respectively. Looking at sperm velocities, neither curvilinear nor straight-line velocities (VCL or VSL) seemed to be affected by any of the TiO_2_ NP concentrations tested. A previous study done by our team also assessed the effect of similar concentrations of TiO_2_ NP in the same species, finding no effects on any concentrations (0, 10–10,000 μg L^−1^) (Carvalhais et al. 2022). Contrarily, in the present study concentrations of 10,000 μg L^−1^ or higher negatively affected the motility. This fact could be explained by the common heterogeneity present in sperm samples that could only be counteracted by increasing the number of analysed samples. Other studies performed in marine organisms also showed evidence of reproductive impairment as consequence of an exposure to TiO_2_ NPs. In the fish *Capoeta trutta* and in the bivalve *Tegillarca granosa*, sperm direct exposure to TiO_2_ NP (10 to 10, 100 μg L^−1^) induced a general decrease in different motility parameters (VCL, VSL and VAP) after 2-h exposure (Han et al. 2019; Özgür et al. 2020). These effects are not restricted to male gametes, as in vivo studies also demonstrated changes in the reproductive outcomes of females. In zebrafish (*Danio rerio*), after a chronic TiO_2_ NP exposure of females to 0.1 and 1 mg L^−1^ (100 and 1000 μg L^−1^), there was a significant reduction in the number of eggs after 13 weeks, suggesting a defect in folliculogenesis. Actually, these authors also described a developmental block of follicular maturation and a significant increase in TiO_2_ NP accumulation in the ovaries of these fish (Jiangxin Wang et al. 2011). Taking together these studies and our results, we may suggest both an in vivo and in vitro reprotoxic effect of supra-environmental concentrations of TiO_2_ NPs in marine organisms.

Exposure of sperm to increasing concentrations of Ag NP and Ag^+^ showed adverse effects on motility. While Ag NP diminished TM and PM for all concentrations, Ag+ only impaired TM at the highest concentration (250 μg L^−1^) and PM after exposure to 0.25 and 250 μg L^−1^. Velocity parameters were affected only after exposure to the highest concentration of Ag NP and Ag+. However, Ag NP affected VCL and VSL, while Ag^+^ exclusively depleted VCL. The susceptibility of VCL to Ag NP was previously suggested in Carvalhais et al. (2022) since VCL decreased in the 250 μg L^−1^ (highest) concentration versus 25 μg L^−1^, despite both being similar to the control. Once again, the heterogeneity between sperm samples obligates a considerable number of samples used in this type of studies, to confirm a reprotoxic effect. The effect of both Ag NP and Ag^+^ in the reproduction of marine organisms is poorly studied so far. Nonetheless, a similar effect in spermatozoa was observed in humans (Enyin Wang et al. 2017; Moretti et al. 2013) and other mammals (Shehata et al. 2021; Olugbodi et al. 2020), where the exposure to the highest concentrations of Ag NP decreased sperm motility parameters in general, showing a transversal reprotoxic effect of these NPs across taxa.

To overcome this high variability commonly observed in sperm samples, another approach currently used in the analysis of sperm quality is the study of fish sperm subpopulations, which considers the results of the kinetic parameters individually, cell by cell. The appearance of sperm subpopulations means that sperm is not homogenous throughout the whole population of an ejaculate and that spermatozoa with various kinematic properties exist within one sample (Martínez-Pastor 2022). These spermatozoa may represent different susceptibilities to treatments or toxins. In this study, we identified 4 distinct clusters potentially representing four sperm subpopulations. The number of subpopulations could vary inter and intra-species, but the scarce reports published on fish showed that sperm samples display three to four subpopulations (Marinović et al. 2021). In that sense, spermatozoa from some species such as *C. macroporum* (Gallego et al. 2017), *S. aurata* (Beirão et al. 2011), *O. mikiss* (Kanuga et al. 2012) and *A. anguilla* (Gallego et al. 2015) showed three sperm subpopulations, while four sperm subpopulations have been identified and described in *S. senegalensis* (Martínez-Pastor et al. 2008) or *G. aculeatus* (Comber et al. 2004). The two studies (ours and Beirão et al. 2011) for gilthead seabream sperm subpopulations provided contrasting results, as Beirão et al. (2011) only identified 3 different sperm subpopulations, without identification of the fast and non-linear SP. These could be due to differences in broodstocks (either at physiological or environmental levels), revealing that the number of subpopulations is not species-specific and seems more related to fish conditions. In addition to the number of subpopulations, their biological meaning is even more important and could be used for assessing different techniques and processes, such as cryopreservation protocols, toxicity tests, etc. Some authors have correlated the different subpopulations with crucial parameters such as fertilization rates (Gallego et al. 2017; Beirão et al. 2011). Samples containing higher percentages of subpopulations with fast and liner spermatozoa showed higher fertilization rates. In that sense, in the 4 populations described in our study, SP1 and SP2 could be considered the fast ones (with higher probabilities of achieving high fertilization rates), while subpopulations SP3 and SP4 could be considered as the slow ones, with lower chances of obtaining fertilization.

In the present study, the reprotoxic effect of both TiO_2_ NP and AgNP/Ag^+^ at the level of sperm subpopulations was evident. The percentage of the fastest subpopulations, SP1 and SP2, were only above 50% (53.4%) in the control group and decreased after exposure to all concentrations of all NPs tested. This decrease was minimal for the Ag^+^ concentrations. However, the most pronounced decrease was for supra-environmental levels of TiO_2_ NP of (1000 μg L^−1^) and Ag NP (250 μg L^−1^). In this last case, the percentage of SP1 and SP2 decreased to 35%, coinciding with the significant decrease in both velocities observed previously for this concentration of Ag NP. To the best of our knowledge, no previous studies have tested the differential effect of NPs in different sperm subpopulations. However, the previously mentioned study by Beirão et al. (2011) has tested the toxicity of cryoprotectants, describing also different effects. In this study, sperm samples were pooled, which is an advantage when few samples are available (low *n*), diminishing sperm heterogeneity. Different cryoprotectants influenced the overall population structure, decreasing spermatozoa velocity and linearity (Beirão et al. 2011). Possibly, some of the cryoprotectants could be inducing a toxic effect in the fastest subpopulation, the one with a higher fertilization hypothesis. Such effects were only detected using the subpopulations approach and would have been missed analysing only motility parameters in a classical fashion.

Contaminants seem to have a clear impact on sperm movement; nevertheless, motility impairment is probably a final output of other physiological alterations induced in the cell. Sperm motility is the most utilized biomarker for the quality of fish spermatozoa, because it is very easy to measure, and movement patterns are directly related to the fertilization capability of spermatozoa (Gallego and Asturiano 2019). Yet, the movement of the spermatozoa is a result of other physiological parameters of the cell (Cosson 2019), thus a reduction in motility parameters implies a general weakening in sperm quality. In the previous study by Carvalhais et al. (2022), TiO_2_ NPs induced a decrease in all the antioxidants for all concentrations and Ag NP–induced superoxide dismutase depletion at the lowest and intermediate concentrations. This impairment of the antioxidant status of gilthead seabream sperm after exposure to NPs could explain the decrease in motility obtained in the present study. However, more research would be needed, to ascertain as well if TiO_2_ NP and Ag NPs enter sperm cells or if their effect is exerted outside through plasma membrane interaction or by interfering in the properties of the activation media. Considering the agglomeration observed for both NPs in suspension (Carvalhais et al. 2022), we may hypothesise that they do not enter the cell. The observed effects could be explained by the secondary toxicity reported by Federici et al. (2007), related to the oxidizing ability of TiO2 NPs, which may lead to the formation of ROS and oxidative stress, this inducing toxicity.

Taking together all the above, and the fact that the fastest sperm subpopulations were reduced by the reprotoxic effect induced by NPs, we may suggest that in contaminated natural environments, natural reproduction, and consequently progeny, could be affected. TiO2 affected sperm total motility only at supra-environmental concentrations, without affecting sperm velocity, while Ag NP produced a negative effect in motility under all concentrations, including realistic ones. For Ag NP, sperm velocity was only impaired when using supra-environmental concentrations. In conclusion, we may say that the reprotoxic effect produced by the NPs under study was more evident under supra-environmental levels, thus fish reproduction could be compromised if the environmental levels of these contaminants increase.

## Data Availability

All data generated or analysed during this study are included in this published article.

## References

[CR1] Asztemborska M, Jakubiak M, Stęborowski R, Chajduk E, Bystrzejewska-Piotrowska G (2018) Titanium dioxide nanoparticle circulation in an aquatic ecosystem. Wat Air and Soil Poll. 229:208. 10.1007/s11270-018-3852-8 10.1007/s11270-018-3852-8 PMC5997115 29950745

[CR2] Beirão J, Cabrita E, Pérez-Cerezales S, Martínez-Páramo S, Herráez MP (2011) Effect of cryopreservation on fish sperm subpopulations. Cryobiology 62:22–31. 10.1016/j.cryobiol.2010.11.005 21112321 10.1016/j.cryobiol.2010.11.005

[CR3] Cabrita E, Ma S, Diogo P, Martínez-Páramo S, Sarasquete C, Dinis MT (2011) The influence of certain aminoacids and vitamins on post-thaw fish sperm motility, viability and DNA fragmentation. Anim Reprod Sci 125:189–195. 10.1016/j.anireprosci.2011.03.003 21482049 10.1016/j.anireprosci.2011.03.003

[CR4] Caldeira C, García-Molina A, Valverde A, Bompart D, Hassane M, Martin P et al (2018) Comparison of sperm motility subpopulation structure among wild anadromous and farmed male Atlantic salmon (*Salmo salar*) parr using a CASA system. Reprod Fertil Dev 30:897–906. 10.1071/rd17466 29650061 10.1071/RD17466

[CR5] Carvalhais A, Oliveira IB, Oliveira H, Oliveira CCV, Ferrão L, Cabrita E et al (2022) *Ex vivo* exposure to titanium dioxide and silver nanoparticles mildly affect sperm of gilthead seabream (*Sparus aurata*) - a multiparameter spermiotoxicity approach. Mar Pollut Bull 177:113487. 10.1016/j.marpolbul.2022.113487 35245769 10.1016/j.marpolbul.2022.113487

[CR6] Comber SCL, Faulkes CG, Look KJWV, Holt WV, Smith C (2004) Recovery of sperm activity after osmotic shock in the three-spined stickleback: implications for pre-oviposition ejaculation. Behaviour 141:1555–1569 http://www.jstor.org/stable/4536223

[CR7] Cosson J (2019) Fish sperm physiology: structure, factors regulating motility, and motility evaluation. In: Yusuf B (Ed.) Biological Research in Aquatic Science (p. Ch. 2). Rijeka: IntechOpen. 10.5772/intechopen.85139

[CR8] Deycard VN, Schäfer J, Petit JCJ, Coynel A, Lanceleur L, Dutruch L et al (2017) Inputs, dynamics and potential impacts of silver (Ag) from urban wastewater to a highly turbid estuary (SW France). Chemosphere 167:501–511. 10.1016/j.chemosphere.2016.09.154 27756044 10.1016/j.chemosphere.2016.09.154

[CR9] Federici G, Shaw BJ, Handy RD (2007) Toxicity of titanium dioxide nanoparticles to rainbow trout (*Oncorhynchus mykiss*): gill injury, oxidative stress, and other physiological effects. Aquat toxicol 84:415–430. 10.1016/j.aquatox.2007.07.009 17727975 10.1016/j.aquatox.2007.07.009

[CR10] Gallego V, Asturiano JF (2018) Sperm motility in fish: technical applications and perspectives through CASA-Mot systems. Reprod Fertil Dev 30:820–832. 10.1071/RD17460 29518349 10.1071/RD17460

[CR11] Gallego V, Asturiano JF (2019) Fish sperm motility assessment as a tool for aquaculture research: a historical approach. Rev Aquac 11:697–724. 10.1111/raq.12253

[CR12] Gallego V, Cavalcante SS, Fujimoto RY, Carneiro PCF, Azevedo HC, Maria AN (2017) Fish sperm subpopulations: changes after cryopreservation process and relationship with fertilization success in tambaqui (*Colossoma macropomum*). Theriogenology 87:16–24. 10.1016/j.theriogenology.2016.08.001 27616215 10.1016/j.theriogenology.2016.08.001

[CR13] Gallego V, Pérez L, Asturiano JF, Yoshida M (2014) Sperm motility parameters and spermatozoa morphometric characterization in marine species: A study of swimmer and sessile species. Theriogenology 82:668–676. 10.1016/j.theriogenology.2014.05.026 25016411 10.1016/j.theriogenology.2014.05.026

[CR14] Gallego V, Vílchez MC, Peñaranda DS, Pérez L, Herráez MP, Asturiano JF et al (2015) Subpopulation pattern of eel spermatozoa is affected by post-activation time, hormonal treatment and the thermal regimen. Reprod Fertil Dev 27:529–543. 10.1071/rd13198 25402273 10.1071/RD13198

[CR15] García-Herreros M (2016) Sperm subpopulations in avian species: a comparative study between the rooster (*Gallus domesticus*) and Guinea fowl (*Numida meleagris*). Asian J Androl 18:889–894. 10.4103/1008-682x.188448 27751988 10.4103/1008-682X.188448 PMC5109881

[CR16] Gil Anaya MC, Calle F, Pérez CJ, Martín-Hidalgo D, Fallola C, Bragado MJ et al (2015) A new Bayesian network-based approach to the analysis of sperm motility: application in the study of tench (*Tinca tinca*) semen. Andrology 3:956–966. 10.1111/andr.12071 26227070 10.1111/andr.12071

[CR17] Han Y, Shi W, Rong J, Zha S, Guan X, Sun H et al (2019) Exposure to waterborne nTiO2 reduces fertilization success and increases polyspermy in a bivalve mollusc: a threat to population recruitment. Environ Scie Technol 53:12754–12763. 10.1021/acs.est.9b03675 10.1021/acs.est.9b03675 31596577

[CR18] Kanuga MK, Drew RE, Wilson-Leedy JG, Ingermann RL (2012) Subpopulation distribution of motile sperm relative to activation medium in steelhead (*Oncorhynchus mykiss*). Theriogenology 77:916–925. 10.1016/j.theriogenology.2011.09.020 22225678 10.1016/j.theriogenology.2011.09.020

[CR19] Keller AA, McFerran S, Lazareva A, Suh S (2013) Global life cycle releases of engineered nanomaterials. J Nanop Res 15:1692. 10.1007/s11051-013-1692-4

[CR20] Khosravi-Katuli K, Prato E, Lofrano G, Guida M, Vale G, Libralato G (2017) Effects of nanoparticles in species of aquaculture interest. Environ Sci Pollut Res 24:17326–17346. 10.1007/s11356-017-9360-3 10.1007/s11356-017-9360-3 28597390

[CR21] Kurwadkar S, Pugh K, Gupta A, Ingole S (2015) Nanoparticles in the environment: occurrence, distribution, and risks. J Hazard Toxic and Radioact Waste 19:04014039. 10.1061/(ASCE)HZ.2153-5515.0000258

[CR22] Labille J, Slomberg D, Catalano R, Robert S, Apers-Tremelo M-L, Boudenne J-L et al (2020) Assessing UV filter inputs into beach waters during recreational activity: a field study of three French Mediterranean beaches from consumer survey to water analysis. Sci Total Environ 706:136010. 10.1016/j.scitotenv.2019.136010 31855634 10.1016/j.scitotenv.2019.136010

[CR23] Marinović Z, Šćekić I, Lujić J, Urbányi B, Horváth Á (2021) The effects of cryopreservation and cold storage on sperm subpopulation structure of common carp (*Cyprinus carpio* L.). Cryobiology 99:88–94. 10.1016/j.cryobiol.2021.01.007 33450240 10.1016/j.cryobiol.2021.01.007

[CR24] Martínez-Páramo S, Diogo P, Dinis MT, Soares F, Sarasquete C, Cabrita E (2013) Effect of two sulfur-containing amino acids, taurine and hypotaurine in European sea bass (*Dicentrarchus labrax*) sperm cryopreservation. Cryobiology 66:333–338. 10.1016/j.cryobiol.2013.04.001 23583301 10.1016/j.cryobiol.2013.04.001

[CR25] Martínez-Pastor F (2022) What is the importance of sperm subpopulations? Anim Reprod Sci 246:106844. 10.1016/j.anireprosci.2021.106844 34538510 10.1016/j.anireprosci.2021.106844

[CR26] Martínez-Pastor F, Cabrita E, Soares F, Anel L, Dinis MT (2008) Multivariate cluster analysis to study motility activation of *Solea senegalensis* spermatozoa: a model for marine teleosts. Reproduction 135:449–459. 10.1530/rep-07-0376 18367506 10.1530/REP-07-0376

[CR27] Martínez-Pastor F, Tizado EJ, Garde JJ, Anel L, de Paz P (2011) Statistical series: opportunities and challenges of sperm motility subpopulation analysis. Theriogenology 75:783–795. 10.1016/j.theriogenology.2010.11.034 21220164 10.1016/j.theriogenology.2010.11.034

[CR28] Moretti E, Terzuoli G, Renieri T, Iacoponi F, Castellini C, Giordano C et al (2013) In vitro effect of gold and silver nanoparticles on human spermatozoa. Andrologia 45:392–396. 10.1111/and.12028 23116262 10.1111/and.12028

[CR29] Olugbodi JO, David O, Oketa EN, Lawal B, Okoli BJ, Mtunzi F (2020) Silver nanoparticles stimulates spermatogenesis impairments and hematological alterations in testis and epididymis of male rats. Molecules 25. 10.3390/molecules25051063 10.3390/molecules25051063 PMC7179123 32120976

[CR30] Özgür ME, Ulu A, Noma SAA, Özcan İ, Balcıoğlu S, Ateş B et al (2020) Melatonin protects sperm cells of *Capoeta trutta* from toxicity of titanium dioxide nanoparticles. Environ Sci Pollut Res 27:17843–17853. 10.1007/s11356-020-08273-7 10.1007/s11356-020-08273-7 32162220

[CR31] Santolaria P, Soler C, Recreo P, Carretero T, Bono A, Berné JM et al (2016) Morphometric and kinematic sperm subpopulations in split ejaculates of normozoospermic men. Asian J Andrology 18:831–834. 10.4103/1008-682x.186874 10.4103/1008-682X.186874 PMC5109871 27624985

[CR32] Shehata AM, Salem FMS, El-Saied EM, Abd El-Rahman SS, Mahmoud MY, Noshy PA (2021) Zinc nanoparticles ameliorate the reproductive toxicity induced by silver nanoparticles in male rats. Int J Nanomedicine 16:2555–2568. 10.2147/ijn.s307189 33833511 10.2147/IJN.S307189 PMC8020588

[CR33] Valverde A, Castro-Morales O, Madrigal-Valverde M, Camacho M, Barquero V, Soler C et al (2021) Sperm kinematic subpopulations of the American crocodile (*Crocodylus acutus*). PLOS ONE 16:e0248270. 10.1371/journal.pone.0248270 33690716 10.1371/journal.pone.0248270 PMC7942986

[CR34] Wang E, Huang Y, Du Q, Sun Y (2017) Silver nanoparticle induced toxicity to human sperm by increasing ROS (reactive oxygen species) production and DNA damage. Environ Toxicol Pharmacol 52:193–199. 10.1016/j.etap.2017.04.010 28433807 10.1016/j.etap.2017.04.010

[CR35] Wang J, Zhu X, Zhang X, Zhao Z, Liu H, George R et al (2011) Disruption of zebrafish (*Danio rerio*) reproduction upon chronic exposure to TiO2 nanoparticles. Chemosphere 83:461–467. 10.1016/j.chemosphere.2010.12.069 21239038 10.1016/j.chemosphere.2010.12.069

[CR36] Zar JH (1999) Biostatistical Analysis. Prentice-Hall Inc, Upper Saddle River, NJ, USA

[CR37] Zhang C, Hu Z, Deng B (2016) Silver nanoparticles in aquatic environments: Physiochemical behavior and antimicrobial mechanisms. Water Research 88:403–427. 10.1016/j.watres.2015.10.025 26519626 10.1016/j.watres.2015.10.025

